# Weighted Gene Co-expression Network Analysis Identifies CALD1 as a Biomarker Related to M2 Macrophages Infiltration in Stage III and IV Mismatch Repair-Proficient Colorectal Carcinoma

**DOI:** 10.3389/fmolb.2021.649363

**Published:** 2021-04-29

**Authors:** Hang Zheng, Yuge Bai, Jingui Wang, Shanwen Chen, Junling Zhang, Jing Zhu, Yucun Liu, Xin Wang

**Affiliations:** Department of General Surgery, Peking University First Hospital, Beijing, China

**Keywords:** colorectal cancer, microsatellite instability, M2 macrophages, tumor microenvironment, bioinformatics, prognosis

## Abstract

Immunotherapy has achieved efficacy for advanced colorectal cancer (CRC) patients with a mismatch-repair-deficient (dMMR) subtype. However, little immunotherapy efficacy was observed in patients with the mismatch repair-proficient (pMMR) subtype, and hence, identifying new immune therapeutic targets is imperative for those patients. In this study, transcriptome data of stage III/IV CRC patients were retrieved from the Gene Expression Omnibus database. The CIBERSORT algorithm was used to quantify immune cellular compositions, and the results revealed that M2 macrophage fractions were higher in pMMR patients as compared with those with the dMMR subtype; moreover, pMMR patients with higher M2 macrophage fractions experienced shorter overall survival (OS). Subsequently, weighted gene co-expression network analysis and protein–protein interaction network analysis identified six hub genes related to M2 macrophage infiltrations in pMMR CRC patients: *CALD1*, *COL6A1*, *COL1A2*, *TIMP3*, *DCN*, and *SPARC*. Univariate and multivariate Cox regression analyses then determined *CALD1* as the independent prognostic biomarker for OS. *CALD1* was upregulated specifically the in CMS4 CRC subtype, and single-sample Gene Set Enrichment Analysis (ssGSEA) revealed that *CALD1* was significantly correlated with angiogenesis and TGF-β signaling gene sets enrichment scores in stage III/IV pMMR CRC samples. The Estimation of STromal and Immune cells in MAlignant Tumor tissues using Expression data (ESTIMATE) algorithm and correlation analysis revealed that *CALD1* was significantly associated with multiple immune and stromal components in a tumor microenvironment. In addition, GSEA demonstrated that high expression of *CALD1* was significantly correlated with antigen processing and presentation, chemokine signaling, leukocyte transendothelial migration, vascular smooth muscle contraction, cytokine–cytokine receptor interaction, cell adhesion molecules, focal adhesion, MAPK, and TGF-beta signaling pathways. Furthermore, the proliferation, invasion, and migration abilities of cancer cells were suppressed after reducing *CALD1* expression in CRC cell lines. Taken together, multiple bioinformatics analyses and cell-level assays demonstrated that *CALD1* could serve as a prognostic biomarker and a prospective therapeutic target for stage III/IV pMMR CRCs.

## Introduction

Colorectal cancer (CRC) is expected to rank as the third leading incidence of new malignancy and the second for cancer-specific mortality (Bray et al., 2018). While CRC is relatively curable if detected and treated early, approximately 58% of CRCs are diagnosed at progressive and metastatic stages, which pose a grave threat to human health (Siegel et al., 2017). In the past decade, immunotherapy in first-line has achieved robust disease control and durable response for patients with advanced or metastatic mismatch repair-deficient (dMMR) CRCs (Couzin-Frankel, 2013; Diaz et al., 2017; Le et al., 2017; Overman et al., 2017; Morse et al., 2019); this is because dMMR cancers harbor higher numbers of somatic mutations, which result in the increasing generation of aberrant neopeptides, thus facilitating antitumor cytotoxic cell recruitments and immunotherapy responses (Dolcetti et al., 1999; Smyrk et al., 2001; Prall et al., 2004). However, little immunotherapy efficacy was observed in patients with the mismatch repair-proficient (pMMR) subtype (Le et al., 2015), who constitute a substantial proportion (approximately 80 to 85%) of CRCs (Boland et al., 1998; Peltomäki, 2003) and exhibit more unfavorable prognostic outcomes than those with the dMMR subtype (Gryfe et al., 2000; Popat et al., 2005; Malesci et al., 2007). Low mutational burden and lack of capability to recruit antitumor immune cells have been considered as the essential obstacles for pMMR tumors to benefit from immunotherapy (Ganesh et al., 2019). Therefore, alternative immune modulation approaches are eagerly awaited for the majority of CRC patients with the pMMR subtype.

Interactions in the tumor microenvironment (TME) between cancer cells and their surroundings are intricate (Hanahan and Coussens Lisa, 2012). Tumor-associated macrophages (TAMs) are the most abundant immune cells in the TME (up to 50%) and are generally categorized into M1 and M2 subtypes (Najafi et al., 2019). M1 plays a tumoricidal effect through secreting pro-inflammatory cytokines like CXCL9 and CXCL10 and initiating inflammatory response to enhance adaptive immune response at the preliminary oncogenesis stage, while the M2 subtype often releases anti-inflammatory cytokines and contributes to angiogenesis, tumor progression, and immunosuppression at the advanced tumor stage (Tan et al., 2018; Ubil et al., 2018; Xiang et al., 2018). Although fractions of M1 macrophages were higher in dMMR CRCs (Narayanan et al., 2019), no significant difference in M2 macrophages infiltration density was found between the pMMR and dMMR subgroups (Bernal et al., 2011; Zhao Y. et al., 2019). Collectively, these data raise constructive guidance regarding the identification of biomarkers related to M2 macrophage infiltrations to weaken the tumorigenic immunoediting activity in pMMR CRC patients.

Nowadays, widespread analysis of high-throughput technologies via advanced computational techniques facilitates the identification of disease-related biomarkers (Fernandes et al., 2020). Weighted gene co-expression network analysis (WGCNA) is a well-documented systematic bioinformatics tool to extract co-related gene modules and then identify hub genes that correlated with clinical traits of interest (Horvath and Dong, 2008; Kakati et al., 2019), and has been widely applied for potential therapeutic target detections in various cancers (Zeng et al., 2020; Zhang et al., 2020; Zhu and Hou, 2020). In this study, based on RNA-seq data acquired from public database, M2 macrophage fractions in stage III/IV pMMR CRC patients were calculated by the CIBERSORT algorithm, and WGCNA was then performed to identify the associated hub genes. By applying Cox regression analyses, CALD1 was identified as an independent prognostic biomarker. We further validated CALD1 for its roles in TME components and tumor progression at both the gene expression and cellular level. We propose CALD1 as a potential target for pMMR CRC for further study.

## Materials and Methods

### Data Pre-processing

The RNA-Seq and corresponding clinical data were acquired from the Gene Expression Omnibus (GEO)^1^ database, including GSE39582 (Marisa et al., 2013) and GSE41258 (Sheffer et al., 2009). GSE39582 was based on the GPL570 platform (Affymetrix Human Genome U133 Plus 2.0 Array), and 200 pMMR stage III/IV CRC samples were utilized as the training group for M2 macrophages-correlated genes selection. The GSE41258 dataset, which was based on GPL96 (Affymetrix Human Genome U133A Array) and contained 82 stage III/IV pMMR CRC samples, was used for external validation. The raw CEL microarray data were downloaded and preprocessed using the RMA method by the “affy” R package (Gautier et al., 2004). If more probes mapped one gene symbol, the maximum value was chosen. The function normalizeBetweenArrays of the “limma” R package was used to achieve consistency between arrays (Ritchie et al., 2015).

### Tumor-Infiltrating Immune Cell (TIIC) Assessment

The CIBERSORT algorithm could accurately quantify the relative infiltration fractions of 22 kinds of immune cells from normalized gene expression profiles by implementing the support vector regression (SVR) machine learning method (Scholkopf et al., 2000; Newman et al., 2015). The TIIC fractions in GSE39582 samples were calculated by “CIBERSORT” R script with the leukocyte gene signature matrix LM22; the significant CIBERSORT *P*-value was set as less than 0.05. The distribution differences of TIICs between dMMR and pMMR tumors were compared by Wilcoxon test, and the associations between M2 macrophage infiltrations and survival status in pMMR patients were investigated.

### Co-expression Network Construction

Stage III/IV pMMR CRC samples with CIBERSORT *p* < 0.05 were screened for WGCNA analysis through the “WGCNA” R package (Langfelder and Horvath, 2008). Generally, the co-expression similarity s_*ij*_ was computed as the absolute value of Pearson’s correlation between nodes i and j:

$$s_{i\,j}=\left|\text{cor}\,\left(x_{i},x_{j}\right)\right|$$

Following this, a weighted adjacency matrix could be calculated by raising s_*ij*_ to a soft thresholding power β, which could intensify strong correlations and reduce weak correlations:

$$a_{i\,j}=s_{i\,j}^{\beta }$$

Next, the adjacency matrix was transformed to a topological overlap matrix (TOM), and highly interconnected genes were clustered into different modules by calculating TOM dissimilarity (1-TOM) with a minimum cluster size of 30 (Yip and Horvath, 2007). Significant module and trait with the highest coefficient was identified for the following analysis. Module eigengene (ME) was defined as the first principal component of the specific module that could represent the module genes. Module membership (MM) was used to determine the correlation between ME and each module, and gene significance (GS) was the Pearson’s correlation between each gene expression and the representative trait. Genes with high intramodular connectivity were regarded as hub genes; the parameters of candidate hub genes were set as MM > 0.8 and GS > 0.2 (Horvath and Dong, 2008).

### Functional and Pathway Enrichment Analysis

Gene Ontology (GO) and Kyoto Encyclopedia of Genes and Genomes (KEGG) pathway enrichment analyses of candidate hub genes were conducted via the “clusterProfiler” R package; an adjusted *p*-value of less than 0.05 was regarded as statistically significant (Yu et al., 2012).

### Protein–Protein Interaction (PPI) Network Integration and Analysis

The PPI network was built by the online STRING database^2^ (Szklarczyk et al., 2019) and mapped in the Cytoscape software (version 3.7.2)^3^ (Shannon et al., 2003). Hub genes were ranked by six methods provided from the cytoHubba plugin (Chin et al., 2014). Then we calculated and visualized Spearman’s correlations between hub gene expressions and M2 macrophage fractions.

### Prognostic Hub Gene Identification and Validation

The prognostic values of the hub genes were assessed by log-rank test on the total of 200 stage III/IV pMMR CRC patients of the GSE39582 dataset. Hub genes as well as clinicopathological factors such as sex, chemotherapy, and the TNM stage were subjected to univariate and multivariate Cox regression analyses to explore independent prognostic factors.

### Tumor Microenvironment Analysis

Firstly, we conducted Spearman correlation analysis for CALD1 expression with 22 types of TIICs based on CIBERSORT results. Then, the consensus molecular subtype (CMS) categorizations of CRC patients were performed via the “CMSCaller” R package (Eide et al., 2017), and the differences in the CALD1 expression between CMS subgroups were analyzed using the Kruskal–Wallis test followed by Dunn *post hoc* tests for multiple comparisons via the “FSA” R package. Next, the Estimation of STromal and Immune cells in MAlignant Tumor tissues using Expression data (ESTIMATE) algorithm was applied via the “estimate” R package to presume the immune and stromal cellular heterogeneity in the TME (Yoshihara et al., 2013). In addition, based on “HALLMARK_ANGIOGENESIS” and “HALLMARK_TGF_BETA_SIGNALING” gene sets extracted from the Molecular Signature Database (MSigDB)^4^, the single sample gene set enrichment analysis (ssGSEA) algorithm was employed via the “GSVA” R package to quantify each sample’s enrichment score, which represents the cumulative enrichment degree of the genes in the assigned gene set in the individual sample (Hänzelmann et al., 2013). Finally, correlations of *CALD1* expression with ssGSEA enrichment scores, immune and stromal scores, as well as immune cell markers were analyzed and visualized via the “ggcorrplot” R package.

### Gene Set Enrichment Analysis (GSEA) of Diverse Pathway Enrichments

Two hundred cases of GSE39582 were separated into CALD1-high and CALD1-low expression groups according to the median *CALD1* expression value. GSEA (Subramanian et al., 2005) was then performed to explore different enriched KEGG pathways between two groups via the “clusterProfiler” R package (Yu et al., 2012), and gene sets with an adjusted *P* < 0.05 was considered as significantly enriched.

### Cell Culture and Transfection

Human pMMR CRC cell lines SW480, SW620, and Caco-2 and dMMR cell line HCT116 (Berg et al., 2017) were obtained from the Cancer Institute of the Chinese Academy of Medical Sciences. SW480, SW620, and Caco-2 cells were cultured in Dulbecco’s modified Eagle’s medium (DMEM, Biological Industries, Israel), and HCT116 was cultured in McCoy’s 5A Medium (Biological Industries, Israel) supplemented with 10% fetal bovine serum (FBS, Biological Industries, Israel) and 1% penicillin–streptomycin (Biological Industries, Israel) at 5% CO_2_ and 37°C.

### Western Blotting (WB)

Total protein lysates of cells were extracted with a RIPA lysis buffer supplemented with 1 mM phenylmethylsulfonyl fluoride (PMSF) and 1 mM protease inhibitor cocktail (Beyotime, Shanghai, China). A BCA assay kit (Beyotime, Shanghai, China) was utilized to measure total protein concentration, and 30 μg of denatured proteins was subjected to 10% SDS-PAGE and electroblotted onto PVDF membranes (Millipore, Burlington, MA, United States). After that, the membranes were blocked with 5% milk in TBST for 1 h at room temperature and then incubated with primary antibodies containing rabbit monoclonal anti-Caldesmon (1:1000, Abcam, Cambridge, MA, United States) and GAPDH (1:1000, Cell Signaling Technology, Danvers, MA, United States) overnight at 4 °C. Finally, the membranes were incubated with corresponding secondary antibodies (1:8000, ZSGB-BIO, Beijing, China) for 1 h at room temperature and then treated with a chemiluminescent HRP substrate (Millipore, Burlington, MA, United States) and exposed to the Bio-Rad imaging system.

### siRNA Transfection

Transient knockdown of *CALD1* in SW480 and SW620 cells was achieved by siRNA transfection. The siRNA sequences for *CALD1* (sense 5′–3′, GGAGGAGAUGCGACUCGAATT) and normal control (NC, sense 5′–3′, UUCUCCGAACGUGUCACGUTT) were synthesized by GenePharma (Jiangsu, China). Cells with confluences of 70–80% were transfected with 100 nM *CALD1* or NC siRNA using GP-siRNA-Mate (GenePharma, Jiangsu, China). After transfection for 48 h, cells were harvested and *CALD1* knockdown was confirmed by WB.

### Cell Migration and Invasion Assay

The metastasis abilities of SW480 and SW620 cells were routinely measured in Transwell chambers (8-μm pore size, Corning, NY, United States). The cells (100,000 cells) were inoculated in a serum-free DMEM on the top chamber, while the bottom chamber contained a 600 μl complete medium. After incubation for an appropriate time, cells on the bottom of the membrane were fixed and stained with 0.1% crystal violet solution. For cell invasion assay, the upper compartment of the chamber was precoated with matrix gel.

### Cell Proliferation Assay

After transfection for 24h, cells were plated into 96-well plates (2,000 cells/well). At the appointed time points (6, 24, 48, and 72 h), the cells were incubated with a 10 μL cell counting kit 8 (CCK8) solution (Bimake, Houston, TX, United States) for additional 2 h. Then, cell viability was determined by recording a 450 nm absorbance value, and comparisons between normal cancer cells, si-NC, and si-CALD1 transfected cells were conducted by Kruskal–Wallis one-way analysis of variance (ANOVA).

### Statistical Analyses

All statistical analyses were performed via R software 3.6.3. Cox regression analysis and Kaplan–Meier log-rank test was performed for survival analysis, and the optimal cut-off value was produced by the “surv_cutpoint” function of the “survminer” R package. All experiments were repeated at least three times, and a *P*-value of <0.05 was deemed statistically significant.

## Results

### TIIC Landscape in Stage III/IV pMMR and dMMR CRC Patients

**FIGURE 1:**
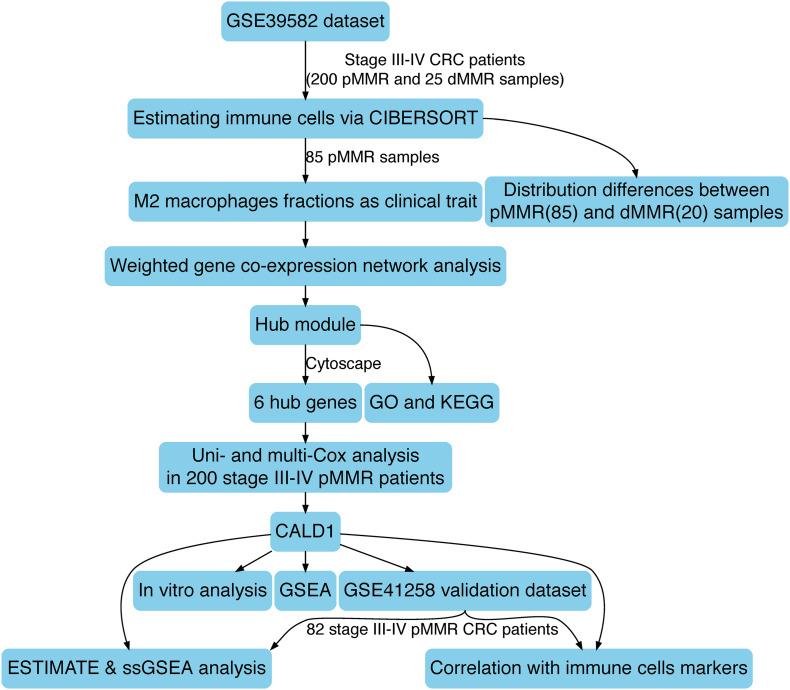
The schematic diagram of this study.

**FIGURE 2:**
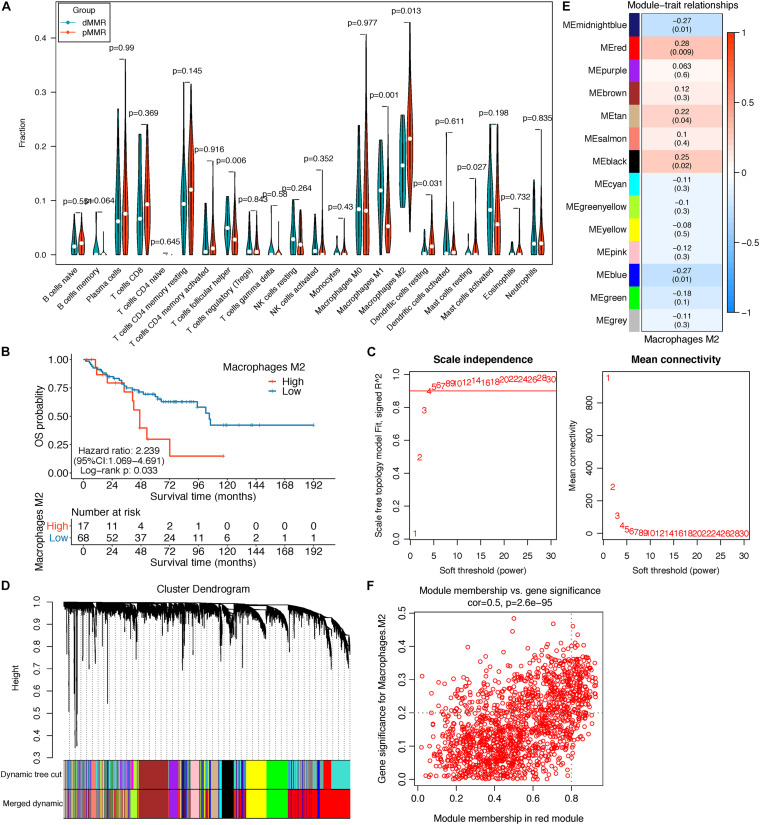
**(A)** Violin plot displayed the CIBERSORT TIIC fraction difference between pMMR (85) and dMMR (20) stage III/IV CRC samples; only samples with a CIBERSORT *P*-value < 0.05 were screened for this analysis. **(B)** Survival curve of the high- and low-M2 macrophage groups in stage III/IV pMMR CRC patients. **(C–F)** Co-expression network analysis by WGCNA. **(C)** Analysis of network topology for optimal soft-threshold power. **(D)** Dendrogram of genes clustered with dissimilarity based on topological overlap; each color below represented an expression module of highly interconnected groups of genes in the constructed gene co-expression network. Gray indicated that genes were not incorporated into any module. **(E)** Heatmap of the correlation between each module’s module eigengene and M2 macrophage fractions; each cell contains the Pearson’s correlation coefficient and the corresponding *P*-value. The red module was the most significant module with the strongest correlation. **(F)** Scatterplot of gene significance (GS) for M2 macrophage fractions vs. module membership (MM) in the red modules. TIICs, tumor-infiltrating immune cells; pMMR, mismatch-repair-proficient; dMMR, mismatch-repair-deficient; CRC, colorectal cancer; WGCNA, weighted gene co-expression network analysis.

The schematic diagram of this research is manifested in Figure 1. Firstly, CIBERSORT identified 85 pMMR and 20 dMMR significant samples (CIBERSORT *P*-value < 0.05), M2 macrophage fractions were higher in pMMR patients (Figure 2A), and pMMR patients with higher M2 macrophage fractions experienced shorter overall survival (OS) (Figure 2B). Subsequently, the M2 macrophage fractions in 85 stage III/IV pMMR CRC patients were chosen as clinical traits for WGCNA construction.

### Co-expression Network Construction and M2 Macrophage Related Gene Identification

The top 5,000 genes with the highest median absolute deviation (MAD) expression values were incorporated for co-expression network construction. To ensure a scale-free network, a soft thresholding power β of 6 was chosen to plant a hierarchical clustering tree (Figure 2C), and 13 gene modules were identified by average linkage clustering (Figure 2D). Then, we calculated the correlations between MEs and M2 macrophage infiltration levels and noticed that the red module exhibited the highest correlation (cor = 0.28, *P* = 0.009) (Figure 2E). Next, we found that the GS for M2 macrophage infiltrations was significantly and positively correlated with MM in the red module (cor = 0.5, *P* = 2.6e-95, Figure 2F). Therefore, the red module was chosen for subsequent analysis.

### GO and KEGG Enrichment Analysis

**FIGURE 3:**
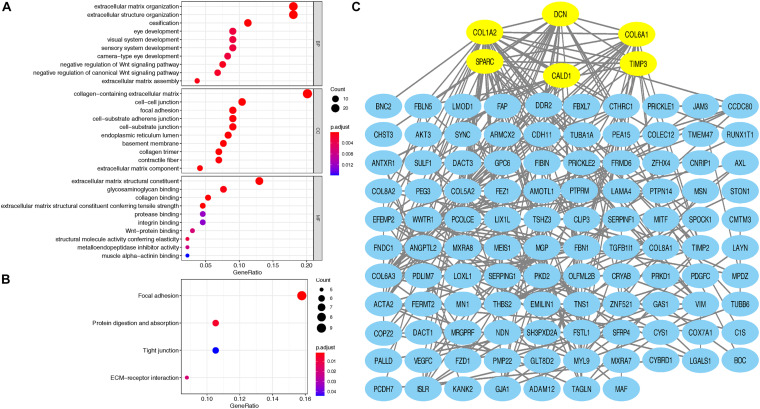
**(A)** Top 10 GO terms, including BP, CC, and MF, respectively, enriched according to the hub genes in the red module; **(B)** KEGG terms enriched according to the hub genes in the red module; **(C)** Cytoscape software was used to establish the PPI network of hub genes in the red module based on the STRING website; yellow signifies the hub nodes. GO, Gene Ontology; BP, biological process; CC, cellular component; MF, molecular function; KEGG, Kyoto Encyclopedia of Genes and Genomes; PPI, protein–protein interaction.

Taking GS > 0.2 and MM > 0.8 as the hub gene thresholds, 144 genes in the red module were screened for GO and KEGG analyses. In the GO analysis, extracellular matrix organization (*P* = 2.60e-13) and extracellular structure organization (*P* = 2.82e-12) were the main enriched biological processes, collagen-containing extracellular matrix (*P* = 2.75e-18) was the major enriched cellular component, and extracellular matrix structural constituent (*P* = 1.01e-12) was the principal enriched molecular function term (Figure 3A). Focal adhesion (*P* = 0.0015), protein digestion and absorption (*P* = 0.0061), ECM–receptor interaction (*P* = 0.019), and tight junction (*P* = 0.0441) were the significantly enriched KEGG pathways (Figure 3B).

### PPI Network Construction and Analysis

**FIGURE 4:**
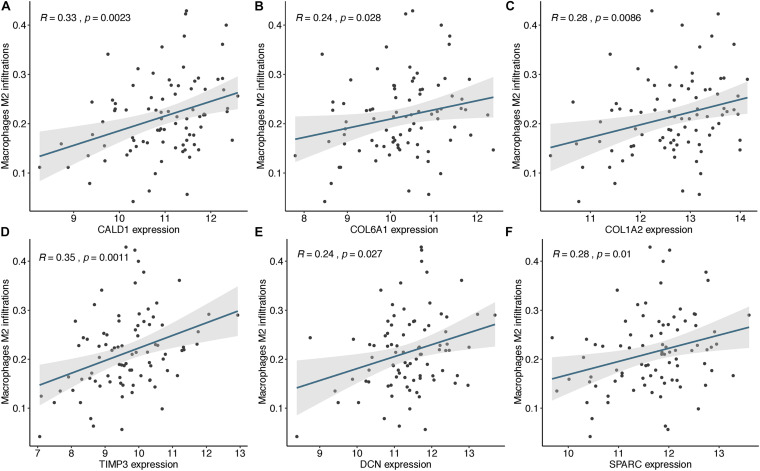
The expressions of CALD1 **(A)**, COL6A1 **(B)**, COL1A2 **(C)**, TIMP3 **(D)**, DCN **(E)**, and SPARC **(F)** were significantly and positively correlated with M2 macrophage infiltrations.

Based on the STRING database, a PPI network was built with the minimum interaction score (median confidence) of 0.4 and was then visualized in the Cytoscape software (Figure 3C). By overlapping the top 15 genes ranked by six methods in cytoHubba, six hub genes were identified for subsequent analysis (Table 1), and Spearman’s correlation analyses revealed that *TIMP3* and *CALD1* expressions were the top two markers positively associated with M2 macrophage fractions (cor = 0.35 and 0.33, respectively) (Figures 4A–F).

**TABLE 1:** Top 15 genes identified by cytoHubba.

Rank	CytoHubba ranking methods					
	
	MNC	Degree	EPC	Closeness	Radiality	Betweenness
1	**COL1A2**	**COL1A2**	COL5A2	**COL1A2**	**COL1A2**	**DCN**
2	COL5A2	COL5A2	**COL1A2**	COL5A2	**SPARC**	WWTR1
3	**SPARC**	**DCN**	**SPARC**	**SPARC**	COL5A2	**COL1A2**
4	FBN1	**SPARC**	**COL6A1**	**DCN**	**COL6A1**	TNS1
5	**DCN**	FBN1	COL6A3	FBN1	**DCN**	**SPARC**
6	**COL6A1**	**COL6A1**	**DCN**	**COL6A1**	FBN1	**CALD1**
7	COL6A3	THBS2	FBN1	THBS2	**CALD1**	AXL
8	THBS2	COL6A3	THBS2	COL6A3	TAGLN	GJA1
9	EFEMP2	**CALD1**	CDH11	**CALD1**	**TIMP3**	CRYAB
10	PCOLCE	EFEMP2	ACTA2	ACTA2	ACTA2	MYL9
11	ACTA2	PCOLCE	EFEMP2	**TIMP3**	THBS2	ISLR
12	FSTL1	ACTA2	**CALD1**	TAGLN	COL6A3	TIMP3
13	CDH11	**TIMP3**	PCOLCE	PCOLCE	FSTL1	TAGLN
14	**TIMP3**	FSTL1	**TIMP3**	FSTL1	VIM	VIM
15	**CALD1**	CDH11	FSTL1	CDH11	CDH11	**COL6A1**

*Symbols in bold type were the genes overlapped by six ranking methods in cytoHubba. MNC, maximum neighborhood component; degree, node connect degree; EPC: edge percolated component.*

### Identification of *CALD1* as an Independent Prognostic Biomarker in Stage III/IV pMMR CRC

**FIGURE 5:**
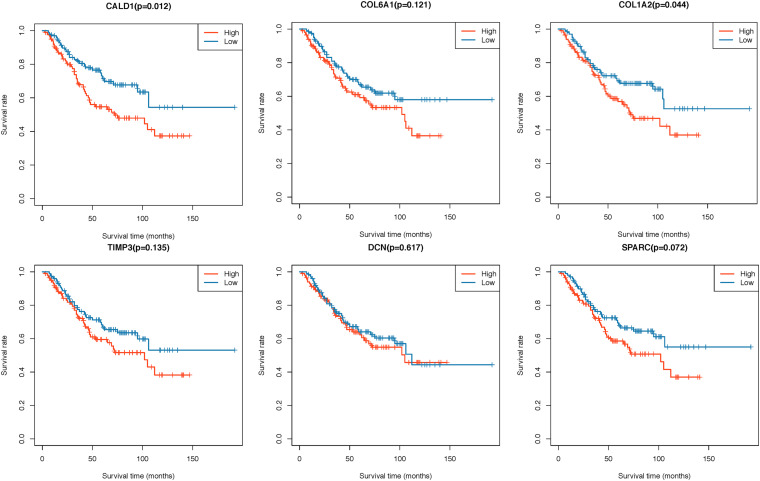
Kaplan–Meier survival curves of six hub genes grouped by their median expression values in the GSE39582 dataset.

Two hundred CRC patients in the GSE39582 cohort were categorized into high/low-expression groups according to the median expression values of each hub gene, then the correlations between the six hub gene expressions and OS were assessed by log-rank test; the results revealed that higher expressions of *CALD1* and *COL1A2* were associated with poorer OS (log-rank *P* = 0.012 and 0.044, respectively) (Figure 5). Subsequently, univariate and multivariate Cox regression analyses were implemented to evaluate the association between hub gene expressions as well as clinicopathological variables and prognosis. As shown in Table 2, age, chemotherapy, tumor stage, *COL1A2*, and *CALD1* were significantly associated with worse prognosis (*P* < 0.05). In multivariate Cox analysis, *CALD1* (HR = 1.868, 95% CI: 1.165–2.995, *P* = 0.009), chemotherapy (HR = 1.869, 95% CI: 1.124–3.109, *P* = 0.016), and tumor stage (HR = 2.658, 95% CI: 1.414–4.996, *P* = 0.002) remained to be significantly correlated with OS. Furthermore, similar analyses were performed in the GSE41258 validation dataset, and multivariate Cox analysis identified that both *CALD1* (HR = 1.859, 95% CI: 1.095–3.158, *P* = 0.022) and tumor stage (HR = 4.951, 95% CI: 2.755–8.929, *P* < 0.001) were independent prognostic predictors (Supplementary Figure 1 and Supplementary Table 1).

**TABLE 2:** Univariate and multivariate Cox proportional hazard regression analysis on OS in GSE39582.

	Univariate analysis			Multivariate analysis		
		
	HR	95% CI	*P*	HR	95% CI	*P*
Age (>65 vs. ≤65)	1.035	1.015–1.055	<0.001	N/A	N/A	0.081
Gender (male vs. female)	1.328	0.838–2.104	0.228			
Location (proximal vs. distal)	1.534	0.969–2.43	0.068	N/A	N/A	0.097
Chemotherapy (no vs. yes)	2.265	1.415–3.625	0.001	1.869	1.124–3.109	0.016
TNM (IV vs. III)	3.559	1.989–6.367	<0.001	2.658	1.414–4.996	0.002
SPARC (high vs. low)	1.52	0.959–2.408	0.075	N/A	N/A	0.609
COL1A2 (high vs. low)	1.601	1.008–2.543	0.046	N/A	N/A	0.975
CALD1 (high vs. low)	1.811	1.131–2.901	0.013	1.868	1.165–2.995	0.009
DCN (high vs. low)	1.123	0.711–1.771	0.619			
COL6A1 (high vs. low)	1.434	0.906–2.27	0.124			
TIMP3 (high vs. low)	1.416	0.895–2.241	0.138			

*OS, overall survival; HR, hazard ratio; CI, confidence interval; N/A, not applicable.*

### *CALD1* Was Correlated With Diverse Tumor Immune Cell Subtypes

**FIGURE 6:**
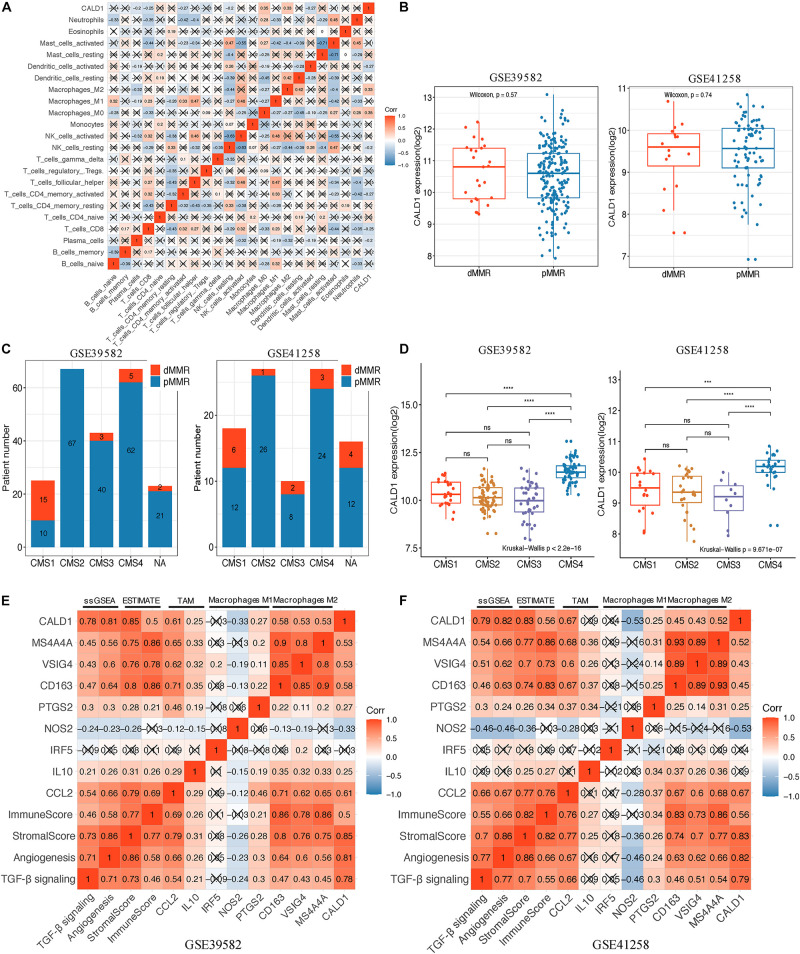
**(A)** Spearman correlation matrix of CALD1 expression with the 22 tumor-infiltrating immune cell proportions in 85 stage III/IV pMMR CRC samples of GSE39582. **(B)** Boxplots of CALD1 expression between dMMR and pMMR stage III/IV CRC samples in both GSE39582 (left) and GSE41258 (right) datasets. **(C)** Histograms of the distribution characteristics of stage III/IV dMMR and pMMR CRC patients in each CMS subgroup. NA, not assigned. **(D)** Boxplots exhibiting CALD1 expression was significantly and specifically upregulated in the CMS4 subtype in both GSE39582 (left) and GSE41258 (right) datasets. Significant differences between CMS subgroups were indicated as follows: ns, not significant; ***P < 0.001, ****P < 0.0001 (Kruskal–Wallis test followed by Dunn’s tests). **(E,F)** Correlation heatmap revealed that CALD1 expression was significantly and positively correlated with angiogenesis and TGF-β signaling ssGSEA enrichment scores, immune and stromal scores, gene markers of TAMs (CCL2 and IL10), and M2 macrophages (CD163, VSIG4, and MS4A4A) but was not clear with gene markers of macrophage M1 (IRF5, NOS2, and PTGS2) in the GSE39582 **(E)** and GSE41258 **(F)** datasets. pMMR, mismatch-repair-proficient; dMMR, mismatch-repair-deficient; CRC, colorectal cancer; CMS, consensus molecular subtypes; TAMs, tumor-associated macrophages.

After identifying the prognostic value of *CALD1* in stage III/IV pMMR CRC patients, we examined the correlations between *CALD1* and TIICs as well as the biomarkers of various immune cells. Figure 6A revealed that *CALD1* was significantly and positively correlated with fractions of M2 macrophages (cor = 0.33, *P* = 0.001) and M0 macrophages (cor = 0.35, *P* = 0.010), whereas it was negatively correlated with fractions of plasma cells (cor = −0.2, *P* = 0.021), CD8 T cells (cor = −0.25, *P* = 0.003), T cell CD4 memory activated (cor = −0.27, *P* = 0.042), NK cells resting (cor = −0.2, *P* = 0.018), and dendritic cells activated (cor = −0.27, *P* = 0.028). As shown in Table 3, *CALD1* expression was significantly correlated with the biomarkers of monocyte, TAM, M2 macrophages, neutrophils, Treg, and T cell exhaustion in both GSE39582 and GSE41258 datasets (*P* < 0.05).

**TABLE 3:** Correlations between CALD1 and immune cells markers.

Description	Gene marker	GSE39582		GSE41258	
			
		Cor	*P*	Cor	*P*
CD8+ T cell	CD8A	0.072	0.313	0.143	0.199
	CD8B	−0.104	0.143	NA	NA
T cell (general)	CD3D	−0.017	0.816	0.134	0.231
	CD3E	−0.058	0.413	−0.036	0.745
	CD2	0.162	*	0.207	0.063
B cell	CD19	−0.025	0.727	0.053	0.634
	CD79A	−0.081	0.253	0.185	0.097
Monocyte	CD86	0.537	***	−0.132	0.236
	CD115	0.586	***	0.585	***
TAM	CCL2	0.630	***	0.681	***
	IL10	0.272	**	0.087	0.436
M1 Macrophage	NOS2	−0.338	***	−0.499	***
	IRF5	−0.033	0.645	−0.051	0.646
	PTGS2	0.246	**	0.252	*
M2 Macrophage	CD163	0.564	***	0.474	***
	VSIG4	0.521	***	0.459	***
	MS4A4A	0.529	***	0.573	***
Neutrophils	CEACAM8	−0.145	*	−0.364	**
	ITGAM	0.640	***	0.577	***
	CCR7	−0.035	0.618	0.115	0.305
Natural killer cell	KIR2DL1	−0.266	**	−0.037	0.740
	KIR2DL3	−0.254	**	−0.270	*
	KIR2DL4	−0.199	**	−0.038	0.737
	KIR3DL1	0.066	0.355	0.371	**
	KIR3DL3	−0.279	***	−0.040	0.721
Dendritic cell	HLA-DPB1	0.486	***	NA	NA
	HLA-DQB1	0.352	***	NA	NA
	HLA-DRA	0.422	***	NA	NA
	HLA-DPA1	0.385	***	NA	NA
	CD1C	0.094	0.187	−0.236	*
	NRP1	0.754	***	0.742	***
	ITGAX	0.421	***	0.312	**
Th1	TBX21	−0.032	0.654	−0.086	0.444
	STAT4	0.297	***	−0.279	*
	STAT1	0.406	***	0.281	*
	IFNG	0.047	0.511	−0.308	**
	TNF	0.073	0.301	−0.307	**
Th2	GATA3	0.315	***	−0.031	0.784
	STAT6	−0.210	**	0.070	0.534
	STAT5A	0.042	0.558	0.124	0.265
	IL13	−0.215	**	−0.219	*
Tfh	BCL6	0.585	***	0.705	***
	IL21	0.074	0.295	−0.272	*
Th17	STAT3	0.034	0.633	−0.002	0.984
	IL17A	−0.067	0.349	−0.370	**
Treg	FOXP3	−0.094	0.185	−0.400	**
	CCR8	−0.076	0.282	−0.132	0.236
	STAT5B	0.264	**	0.268	*
	TGFB1	0.598	***	0.502	***
T cell exhaustion	PDCD1	−0.270	**	−0.303	**
	CTLA4	−0.319	***	−0.135	0.226
	HAVCR2	0.390	***	NA	NA
	GZMB	−0.016	0.826	0.033	0.766

*p < 0.05; **p < 0.01; ***p < 0.0001. Cor, Pearson’s correlation value; TAM, tumor-associated macrophage; Th, T helper cell; Tfh, follicular helper T cell; Treg, regulatory T cell; NA, not available.

### *CALD1* Was Upregulated in CMS4 Subtype and Positively Correlated With Stromal and Immune Scores and Angiogenesis and TGF-β Signaling Enrichment Scores

We incorporated dMMR stage III/IV CRC patients and applied comparative analysis of *CALD1* expression in different CRC subgroups. As shown in Figure 6B, the *CALD1* expression between dMMR and pMMR CRC samples had no significant difference in the two datasets (Wilcoxon test, *P* = 0.57 and 0.74, respectively). Subsequently, we performed CMS analysis via the “CMSCaller” R package to divide CRCs of GSE39582 and GSE41258 into four biologically distinct classifications: CMS1 (dMMR-like immune), CMS2 (canonical), CMS3 (metabolic), and CMS4 (mesenchymal) (Guinney et al., 2015). The majority of dMMR samples were categorized as CMS1 (Figure 6C), and *CALD1* expression was significantly and specifically upregulated in the CMS4 subtype, which is characterized by TGF-β signaling activation, angiogenesis, and stromal invasion (Guinney et al., 2015).

Those results implied the potential regulation mechanisms of *CALD1* in CRC prognosis and TAM recruitments as well as polarizations, and as both intrinsic tumor features and extrinsic tumor microenvironment engage in tumor progression and macrophage polarization (Chen et al., 2019), we subsequently calculated the stromal and immune scores as well as the angiogenesis and TGF-β signaling gene sets enrichment scores of each stage III/IV pMMR CRC sample via the ESTIMATE and ssGSEA algorithms. Consistent with previous researches, higher stromal scores were associated with worse overall survival (GSE39582, log-rank *P* = 0.004; GSE41258, log-rank *P* = 0.138; Supplementary Figures 2A,C), while immune scores had non-significant impact on survival status (GSE39582, log-rank *P* = 0.108; GSE41258, log-rank *P* = 0.578; Supplementary Figures 2B,D) (Liu et al., 2020). The expression of *CALD1* exhibited strong positive correlations with stromal and immune scores in both GSE39582 (cor = 0.85, *P* < 0.001 and cor = 0.5, *P* < 0.001, respectively) (Figure 6E) and GSE41258 (cor = 0.83, *P* < 0.001 and cor = 0.56, *P* < 0.001, respectively) (Figure 6F). The angiogenesis and TGF-β signaling ssGSEA enrichment scores exhibited consistently significant and strong positive correlations with *CALD1* and macrophage marker expressions as well as stromal and immune scores in both GSE39582 and GSE41258 datasets (Figures 6E,F). The complicated interactions between CALD1, macrophage markers, as well as stromal and immune scores indicated that *CALD1* might engage in the complex modulation of macrophage activations across TME alternations, possibly through promoting angiogenesis and activating TGF-β signaling pathways.

### GSEA of *CALD1* Related Signaling Pathways

**FIGURE 7:**
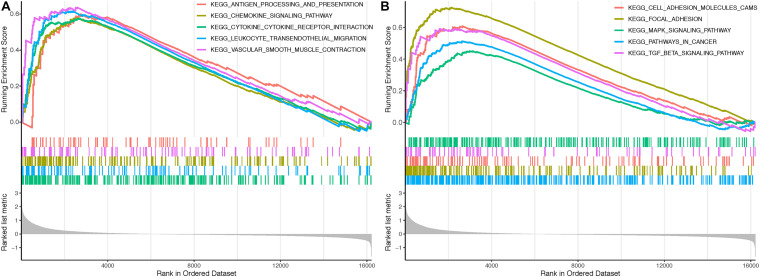
Gene Sets Enrichment Analysis (GSEA) revealed that several immune-related **(A)** and tumor-related **(B)** pathways were highly enriched in the high CALD1 expression group.

Gene Set Enrichment Analysis identified several immune-related and oncogenic KEGG pathways that were enriched in the high-*CALD1* expression group (Figures 7A,B), including antigen processing and presentation, chemokine signaling, cytokine–cytokine receptor interaction, leukocyte transendothelial migration, vascular smooth muscle contraction, cell adhesion molecules, focal adhesion, MAPK signaling pathway, pathways in cancer, and TGF-beta signaling pathway.

### Downregulation of *CALD1* Weakened the Proliferation, Migration, and Invasion in CRC Cells

**FIGURE 8:**
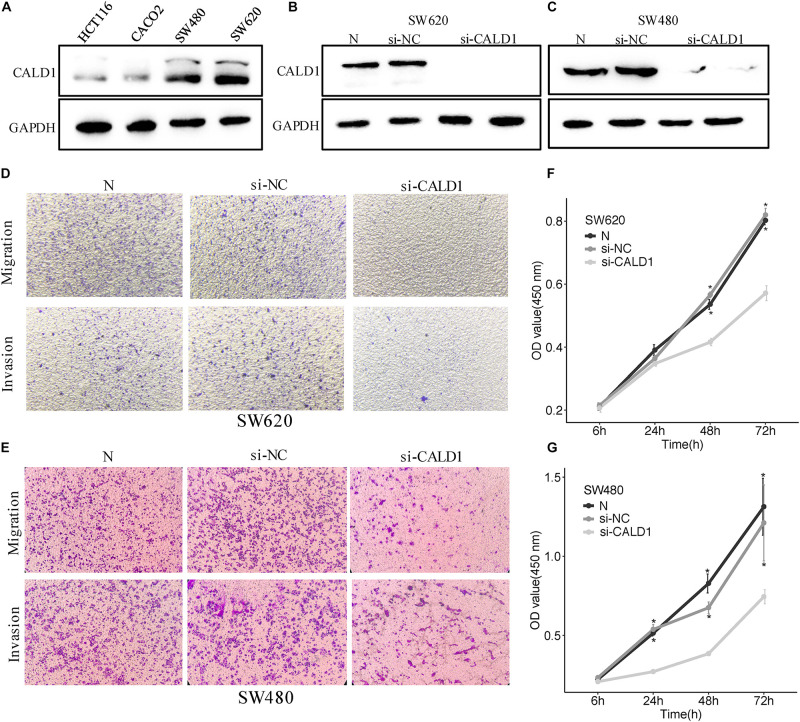
CALD1 promotes cell proliferation, migration, and invasion in CRC cells. **(A)** The protein levels of CALD1 in four kinds of CRC cell lines were measured by Western blot. **(B,C)** The protein levels of normal, si-NC, and si-CALD1 transfected SW620 **(B)** and SW480 **(C)** cells by Western blot; si-CALD1 transfections were performed in duplicate. **(D,E)** Transwell assays revealed that *CALD1* knockdown attenuated SW620 **(D)** and SW480 **(E)** cell migration and invasion. **(F,G)** CCK8 assays were performed to assess cell viability in normal, si-NC, and si-CALD1 transfected SW620 **(F)** and SW480 **(G)** cells. *p < 0.05 in comparison with the si-CALD1 group using Kruskal–Wallis one-way analysis of variance (ANOVA). All assays were repeated at least three times.

To gain a better insight into the value of *CALD1* as a prognostic marker, si-CALD1 transfection was performed to transiently decrease *CALD1* expression in high-*CALD1* expressed SW620 and SW480 cells (Figure 8A), and its efficiency was verified by Western blotting (Figures 8B,C). CCK8 assay revealed that the viability of CRC cells was markedly restrained when *CALD1* was downregulated (Figures 8E,G). Furthermore, silencing *CALD1* conspicuously inhibited the invasion and migration capacities in CRC cells (Figures 8D,F). These findings suggested that *CALD1* could facilitate tumor progression of CRC.

## Discussion

Microsatellite stability accounts for the majority of CRC patients and indicates unfavorable outcomes (Popat et al., 2005). Although immunotherapy has achieved responsiveness in many intractable tumors, no effective immune therapy has demonstrated any benefit for those patients (Le et al., 2015), which inspired us to prioritize the exploration of immune biomarkers to guide immunotherapy applications. As the principal immune subset in the TME, M2 macrophages induce angiogenesis, remodel the matrix, restrain the therapeutic response, and facilitate malignance (Najafi et al., 2019). Hence, approaches that restrain the activity of M2 macrophages would be a compelling area for oncologic research.

In this study, we firstly found significant differences in M2 macrophage distributions between pMMR and dMMR stage III/IV CRC patients, and increased M2 macrophage levels were associated with poorer prognosis for pMMR patients. Then, by applying integrated bioinformatics analysis, we successfully identified *CALD1* as an independent prognostic biomarker closely correlated to M2 macrophage infiltrations. After that, we explored its relationship with diverse TME patterns and signaling pathway functions. In addition, by siRNA-mediated *CALD1* depletion in CRC cell lines, we found that the proliferation and metastasis ability of tumor cells were repressed, which indicated that *CALD1* played a dual role in immunosuppression and tumor metastasis.

Caldesmon (CaD), encoded by *CALD1*, is an actomyosin-binding and cytoskeleton-related protein. There are two major CaD isoforms that evolved through alternative splicing, including high-molecular weight CaD (h-CaD) and low-molecular weight CaD (l-CaD) (Hayashi et al., 1992; Huber, 1997). H-CaD exists uniquely in vascular and visceral smooth muscle cells (SMC) and acts as a cellular contraction regulator and biomarker for SMC-associated neoplasms (Watanabe et al., 1999). L-CaD is widely distributed in non-muscle cells (Sobue and Sellers, 1991; Dabrowska et al., 2004), and studies have shown that l-CaD promoted malignancy in several cancers (Kim et al., 2012; Chang et al., 2013; Lee et al., 2015; Lian et al., 2020). Lee et al. (2015) identified that overexpression of l-CaD in primary non-muscle-invasive bladder cancer is significantly correlated with large tumor size, lymphovascular invasion, advanced stage, higher grade, and adverse prognosis and elucidated that l-CaD-derived morphological changes of tumor cells were the underlying mechanism responsible for enhancing tumor cell motility. Chang et al. (2013) demonstrated that in oral cavity squamous cell carcinoma, the expression of l-CaD was higher in metastatic lymph nodes than in primary tumor cells, and higher l-CaD was associated with tumor metastasis. In CRC, Lian et al. (2020) proved that l-CaD was the isoform derived from alternative splicing of *CALD1* and played a role in tumor metastasis. Kim et al. (2012) reported that the expression of l-CaD was higher in colon cancer than in normal colon mucosa and proposed that l-CaD could be a biomarker to predict neoadjuvant chemoradiotherapy susceptibility. In addition, Zhao et al. performed bioinformatics analysis and identified *CALD1* as candidate genes for the early onset of CRC (Zhao B. et al., 2019). However, until now, the effect of *CALD1* in CRC tumor microenvironment has rarely been addressed.

In the present study, we investigated the association between *CALD1* and M2 macrophage infiltrations at the genomic level by using GEO datasets. As shown in Figure 6A, based on the CIBERSORT results, *CALD1* was positively correlated with M0 and M2 macrophage fractions, whereas it was negatively correlated with CD8 T cell fractions, which were the principal components in antitumor immunity. Figures 6E,F revealed that the expression of *CALD1* was positively correlated with TAM marker *CCL2* and M2 macrophage markers (*CD163*, *VSIG4*, and *MS4A4A*). As for *CCL2*, studies have identified that *CCL2* played a vital role in activating and recruiting TAMs, thereby deriving immunosuppressive effects (Mu et al., 2019; Xue et al., 2019). The blockage of *CCL2* resulted in significantly elevated levels of M1 polarization-associated markers and cytokines, whereas M2-associated markers were diminished (Sierra-Filardi et al., 2014). The positive expression of *CALD1* and *CCL2* hinted that *CALD1* might stimulate and polarize TAMs by upregulating *CCL2* expression; however, further research is needed for confirmation.

Although CALD1 expression revealed no significant difference between dMMR and pMMR samples (Figure 6B), we observed that *CALD1* was specifically upregulated in the CMS4 subtype compared with that in CMS1-3 (Figure 6D). CMS4 has been characterized by the notable upregulation of genes correlated with angiogenesis, TGF-β signaling activation, stromal infiltration, and worse prognosis (Guinney et al., 2015). Given the previous literature on the crosstalk between angiogenesis and TGF-β in macrophage recruitments and M2 polarizations (Allavena et al., 2008; Erreni et al., 2011; Najafi et al., 2019; Rahma and Hodi, 2019), we investigated if *CALD1* correlated with the enrichments for the two gene sets in stage III/IV pMMR CRC samples. Indeed, *CALD1* expression significantly correlated with ssGSEA scores for angiogenesis and TGF-β signaling gene lists (Figures 6E,F), suggesting the prospective synergistic roles of *CALD1* in macrophage recruitments and polarizations as well as driving CRC progression and metastasis. In addition, accumulated evidence has revealed that the complex stromal medium is the core component of TME, and virtually every property of cancer-associated stroma plays a demonstrable role in sustaining hyperproliferation of tumor cells (Tsujino et al., 2007; Ueno et al., 2017; Liu et al., 2020) as well as therapy resistance in one condition or another (Gonçalves-Ribeiro et al., 2017). Our results revealed that stromal score was strongly and positively correlated with *CALD1* (cor = 0.85 and 0.82, *P* < 0.001) and *CCL2* (cor = 0.81 and 0.79, *P* < 0.001) expressions in the two datasets, indicating that signals from intricate *CALD1*, *CCL2*, and stromal interactions might drive M2 macrophage activation and polarization. Furthermore, GSEA disclosed that high expression of *CALD1* was correlated with several immunoregulation pathways associated with M2 macrophage activation and recruitment, such as cytokine–cytokine receptor interaction, leukocyte transendothelial migration, vascular smooth muscle contraction, TGF-beta signaling pathway, and MAPK signaling pathway (Jiang et al., 2017; Wongchana et al., 2018).

Several limitations still exist in our study. Firstly, although functional and enrichment analyses were performed, the specific mechanisms of how *CALD1* activated and recruited M2 macrophages require further clarification. In addition, the intratumoral immune and stromal compositions were estimated only through bioinformatics algorithms at molecular levels. The actual relationship between *CALD1* and M2 macrophage infiltrations was not histologically verified in this study. If possible, we will perform additional biochemical and histological analyses to reevaluate the robustness of our findings in the future. Moreover, although our in vitro validation revealed that *CALD1* was highly expressed in pMMR cell lines (SW480 and SW620), comparison between more CRC cell lines proficient and deficient in the MMR pathway will be necessary to clarify the specificity of *CALD1* for pMMR CRC cells. Finally, as our study was based on public transcriptomic datasets, the application of *CALD1* as a prognostic biomarker and potential target must be researched in prospective real-world pMMR CRC patients receiving immunotherapy.

## Conclusion

In conclusion, this study is the first to reveal pMMR CRC patients possessed higher composition of M2 macrophages than dMMR tumors. We then identified *CALD1* as an independent prognostic marker positively correlated with M2 macrophage infiltrations in stage III/IV pMMR CRC via WGCNA and Cox regression analyses. *CALD1* was upregulated specifically in the CMS4 CRC subtype and was significantly correlated with angiogenesis and TGF-β signaling gene sets ssGSEA enrichment scores as well as immune and stromal ESTIMATE scores. In addition, *CALD1* promoted proliferation, invasion, and migration of CRC cells. *CALD1* could serve as an independent prognostic biomarker and a candidate M2 macrophage target for pMMR CRC patients. However, validation in large-scale genomics and functional and prospective clinical trials are still required.

## Data Availability Statement

The original contributions presented in the study are included in the article/Supplementary Material, further inquiries can be directed to the corresponding author/s.

## Author Contributions

HZ, SC, and XW contributed to the conception and design. YB, JW, and JLZ extracted the data from the databases. HZ, JZ, and SC contributed to the data analysis and interpretation. HZ drafted the manuscript. YL and XW revised the manuscript and supervised the entire study. All authors read and approved the final manuscript.

## Conflict of Interest

The authors declare that the research was conducted in the absence of any commercial or financial relationships that could be construed as a potential conflict of interest.

## Footnotes

1 http://www.ncbinlm.nih.gov/geo/

2 https://string-db.org/

3 http://www.cytoscape.org/

4 https://www.gsea-msigdb.org
