# Supplementary material for: Weighted Gene Co-expression Network Analysis Identifies CALD1 as a Biomarker Related to M2 Macrophages Infiltration in Stage III and IV Mismatch Repair-Proficient Colorectal Carcinoma
Source: Front Mol Biosci. 2021 Apr 29;8:649363. doi: 10.3389/fmolb.2021.649363 (PMC8116739; doi:10.3389/fmolb.2021.649363)
Supplement: Supplementary file 1 [file Data_Sheet_1.PDF]

Supplementary Material

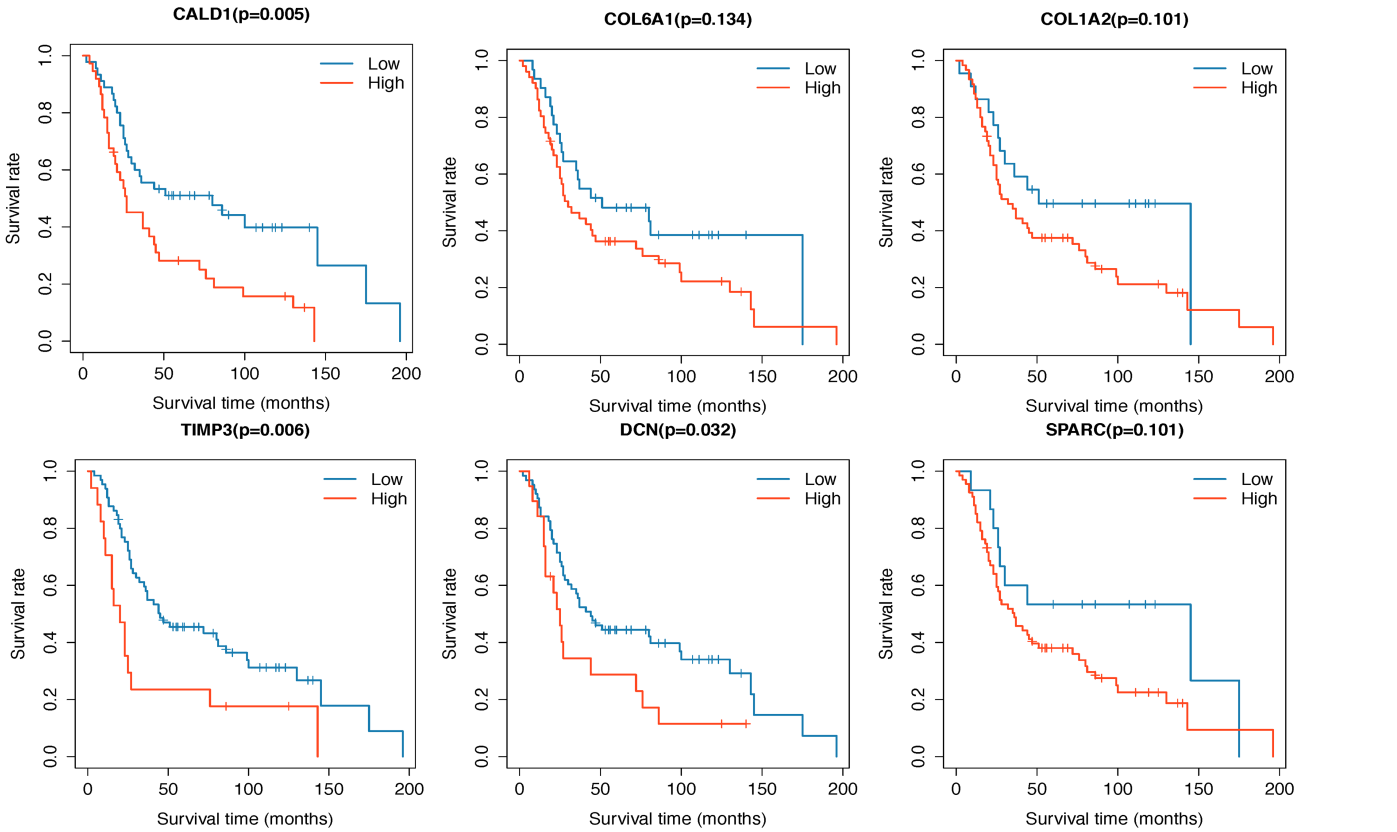

Supplementary Figure 1. Kaplan-Meier survival curves of 6 hub genes grouped by their median expression values in GSE41258 dataset.

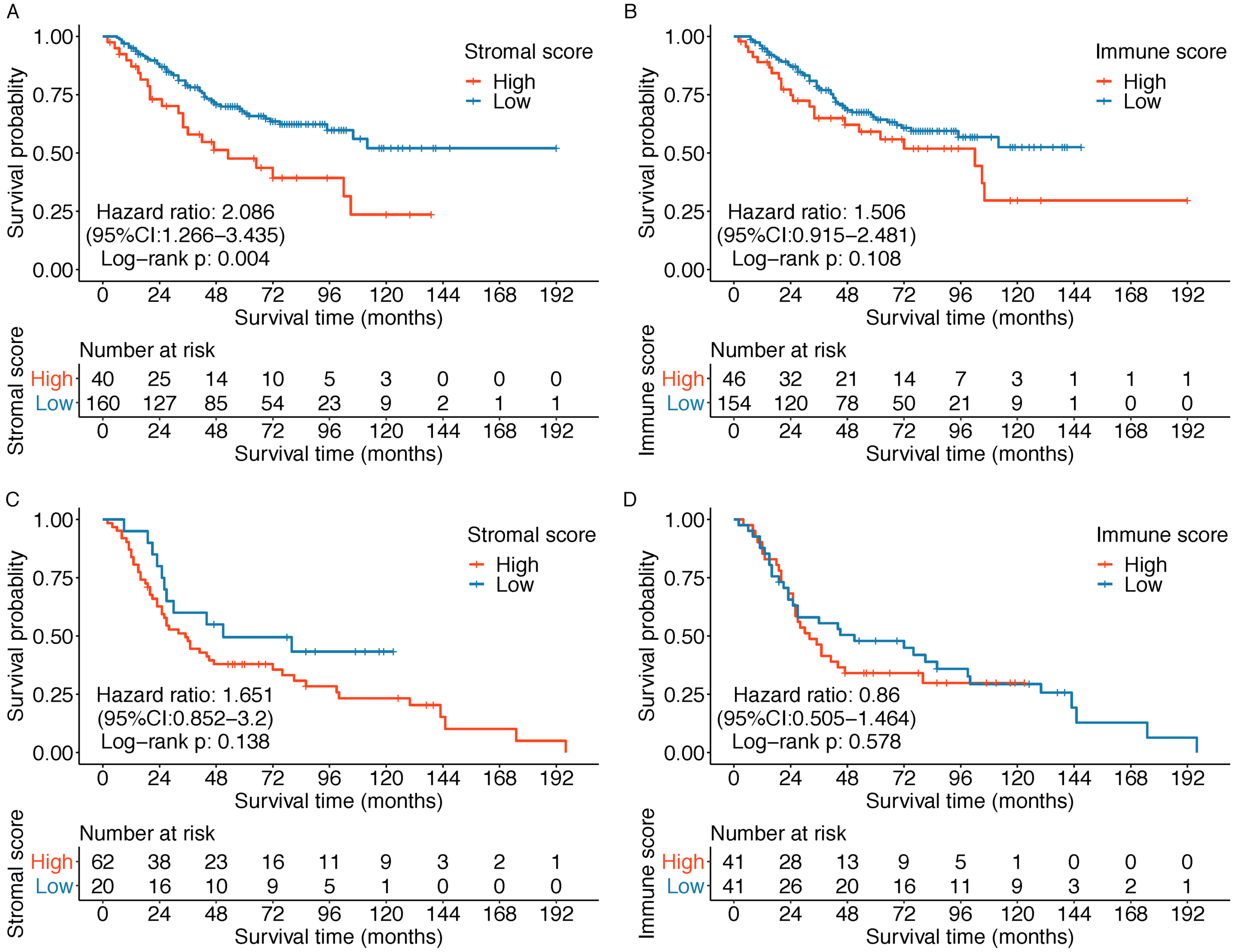

Supplementary Figure 2. Kaplan-Meier survival curves of stromal **(A, C)** and immune **(B, D)** scores by using optimal risk score as cut-off in GSE39582 **(A, B)** and GSE41258 **(C, D)** dataset.

Supplementary Table 1. Univariate and multivariate Cox proportional hazards regression analysis on OS in GSE41258.

	Univariate analysis			Multiivariate analysis		
	HR	95% CI	P	HR	95% CI	P
Age (>65 vs. ≤65)	0.865	0.509-1.471	0.593			
Gender (male vs. female)	1.342	0.798-2.258	0.268			
Location (proximal vs. distal)	1.156	0.639-2.093	0.632			
TNM (IV vs. III)	5.252	2.927-9.424	<0.001	4.951	2.755-8.929	<0.001
SPARC (high vs. low)	1.812	0.88-3.73	0.107			
COL1A2 (high vs. low)	1.693	0.895-3.2	0.105			
CALD1 (high vs. low)	2.091	1.236-3.537	0.006	1.859	1.095-3.158	0.022
DCN (high vs. low)	1.88	1.049-3.368	0.034	N/A	N/A	0.714
COL6A1 (high vs. low)	1.514	0.875-2.618	0.138			
TIMP3 (high vs. low)	2.252	1.242-4.081	0.007	N/A	N/A	0.275

Abbreviations: OS, overall survival; HR, hazard ratio; CI, Confidence interval; N/A, not applicable.
